# Combining the Powerful Antioxidant and Antimicrobial Activities of Pomegranate Waste Extracts with Whey Protein Coating-Forming Ability for Food Preservation Strategies

**DOI:** 10.3390/antiox13111394

**Published:** 2024-11-15

**Authors:** Sara Viggiano, Rita Argenziano, Adriana Lordi, Amalia Conte, Matteo Alessandro Del Nobile, Lucia Panzella, Alessandra Napolitano

**Affiliations:** 1 Department of Chemical Sciences, University of Naples “Federico II”, Via Cintia 6, 80126 Naples, Italy; sara.viggiano@unina.it (S.V.); rita.argenziano@unina.it (R.A.); panzella@unina.it (L.P.); 2 Department of Agricultural Sciences, University of Naples “Federico II”, Carlo di Borbone 1, 80055 Naples, Italy; 3 Department of Economics, Management and Territory, University of Foggia, Via A. da Zara 11, 71122 Foggia, Italy; adriana.lordi@unifg.it (A.L.); matteo.delnobile@unifg.it (M.A.D.N.); 4 Department of Department of Humanistic Studies, Letters, Cultural Heritage, Educational Sciences, University of Foggia, Via Arpi 176, 71121 Foggia, Italy; amalia.conte@unifg.it

**Keywords:** pomegranate peels and seeds, antioxidant, active food packaging, phenolic compounds, antimicrobial activity, lipid peroxidation, enzymatic browning, whey proteins

## Abstract

Different solvents water, ethanol and ethanol/water (6:4 *v*/*v*), were compared in the extraction of pomegranate peels and seeds (PPS) in terms of recovery yields, antioxidant properties, and antimicrobial action against typical spoilage bacterial and fungal species. The best performing extract (ethanol/water (6:4 *v*/*v*) was shown to contain mostly ellagic acid and punicalagin as phenolic compounds (5% overall) and hydrolysable tannins (16% as ellagic acid equivalents) and was able to inhibit the growth of the acidophilic *Alicyclobacillus acidoterrestris* at a concentration as low as 1%. The preservation of the organoleptic profile of *A. acidoterrestris*-inoculated apple juice with extract at 1% over 20 days was also observed thanks to the complete inhibition of bacterial growth, while the extract at 0.1% warranted a significant (40%) inhibition of the enzymatic browning of apple smoothies over the first 30 min. When incorporated in whey proteins’ isolate (WPI) at 5% *w*/*w*, the hydroalcoholic extract conferred well appreciable antioxidant properties to the resulting coating-forming hydrogel, comparable to those expected for the pure extract considering the amount present. The WPI coatings loaded with the hydroalcoholic extract at 5% were able to delay the browning of cut fruit by ca. 33% against a 22% inhibition observed with the sole WPI. In addition, the functionalized coating showed an inhibition of lipid peroxidation of Gouda cheese 2-fold higher with respect to that observed with WPI alone. These results open good perspectives toward sustainable food preservation strategies, highlighting the potential of PPS extract for the implementation of WPI-based active packaging.

## 1. Introduction

Agri-food by-products are nowadays increasingly regarded as a low-cost source of value-added compounds, among which polyphenols prominently appear because of their well-established antioxidant and beneficial health properties [1,2,3,4,5]. A remarkable example is represented by the waste of pomegranate juice production in the form of peels and seeds (PPS), obtained in up to 4.2 million tons per only 1 ton of juice [6].

In addition to anthocyanins, flavonoids, and phenolic acids, pomegranate contains two specific ellagitannins, punicalagin and punicalin, together with ellagic acid that are well known for their beneficial health properties [1,6,7,8,9,10]. In fact, the consumption of pomegranate was reported to have cardio-protective, anti-inflammatory, anti-allergic, and immune function support and cholesterol-lowering effects, together with protective properties against cancer and type 2 diabetes [11,12,13,14].

Most of the bioactive components of pomegranate are present also at high levels in the PPS [15]. Water, ethanol, and methanol were shown as efficient solvents to obtain PPS extracts enriched in phenolic compounds (total phenol content around 300 gallic acid eqs/g) [15]. The PPS extracts exhibited high antioxidant properties, as evaluated by 2,2-diphenyl-1-picrylhydrazyl (DPPH) (ranging from 7 to 9 μmol Trolox eqs./g, depending on the solvent used), ferric-reducing/antioxidant power (FRAP) (1.60 mmol Fe (II)/g), and 2,2′-azino-bis(3-ethylbenzothiazoline-6-sulfonic acid) (ABTS) (11 Trolox eqs/g) assays [15]. Furthermore, the remarkable antimicrobial activity of PPS extracts was documented [16,17]. Thanks to these properties, pomegranate waste extracts were fully exploited as functional additives, e.g., cosmetic formulations or in food applications [18,19].

In this context, it is worth noting that, in the last decades, the use of natural additives has been encouraged, especially in the food packaging sector in which the demand for safe, non-toxic materials that may allow food preservation is perceived as urgent. Proteins- or carbohydrates-based biopolymers that are biodegradable and environmentally safe are considered viable alternatives to synthetic polymers such as polyethylene, polypropylene, or polystyrene, commonly used in food packaging [20,21]. In particular, the large availability of whey protein, as the main by-product of the dairy industry, has aroused increasing attention to the use of this material as a major component for food packaging. Indeed, thanks to the presence of β-lactoglobulin (3.03 g/L) and α-lactalbumin (1.02 g/L), whey proteins are able to form biodegradable and biocompatible edible films and coatings endowed with good technological properties for food applications [22,23].

The possibility to impart biopolymers-based materials functional properties, such as antioxidant and antimicrobial activities, as well as to improve their stability and mechanical properties by the use of polyphenol-rich extracts was also considered [24,25,26]. For example, the addition of pomegranate peel extracts to chitosan films was shown to significantly improve the film mechanical properties, whereas in the case of starch or fish gelatin edible films, an increase in the resistance to microbial growth against *Staphylococcus aureus*, *Salmonella* spp., *Listeria monocytogenes*, and *Escherichia Coli* was observed [27,28].

In this study, we aimed to assess whether the antioxidant and antimicrobial properties of the PPS extracts were preserved after their incorporation into whey protein isolate (WPI)-based coatings and whether the resulting materials could be exploited for preserving different food matrices from deterioration.

Herein, we report the food-grade solvent extraction of low-molecular-weight phenolic compounds from PPS that was optimized based on the determination of the recovery yields, total phenolic content (TPC), and assessment of the antioxidant properties by chemical assays together with the evaluation of antibacterial activity on typical spoilage bacterial and fungal species. The most promising proved to be the hydroalcoholic extract that was subjected to LC-MS analysis for identification and quantitation of the main phenolic components. The ability of this extract to efficiently inhibit the growth of a typical food spoilage bacterium like *A. acidoterrestris* in apple juice and the enzymatic browning in apple smoothies prompted us to explore its use as an active component in coatings obtained from whey protein isolate (WPI) for food packaging applications. Differently from most reports describing the addition of glycerol and/or other plasticizers to prepare the WPI coatings [29], only denatured WPI was used in this study to prepare the functional coatings that proved able to preserve food models against lipid peroxidation and enzymatic browning.

## 2. Materials and Methods

### 2.1. General Experimental Methods

Pomegranates, Red Delicious apples, and Gouda cheese were purchased at a local supermarket. WPI was obtained from commercial sources (MyProtein). DPPH, iron(III) chloride (97%), 2,4,6-tris(2-pirydyl)-s-triazine (TPTZ) (≥98%), (±)-6-hydroxy-2,5,7,8-tetramethylchromane-2-carboxylic acid (Trolox) (97%), Folin–Ciocalteu reagent, gallic acid (≥97.5%), ellagic acid (≥95%), thiobarbituric acid, ammonium iron(II) sulfate ((NH)_4_Fe(SO_4_)_2_6H_2_O, ≥98%), and xylenol orange were obtained from Sigma-Aldrich (Milan, Italy) and used as obtained.

UV-Vis spectra were recorded using a Jasco (Cremella, Lecco, Italy) V-730 spectrophotometer. Colorimetric analyses were performed using the CR-400/410 Konica Minolta (Milano, Italy) Chroma Meter.

HPLC analyses were run using an Agilent instrument (Cernusco sul Naviglio, Milan, Italy) equipped with a UV-Vis detector. A phenomenex Sphereclone ODS column (250 × 4.60 mm, 5 µm) (Castel Maggiore, Bologna, Italy) was used.

The following elution was used: 0.1% formic acid (solvent A)/methanol; (solvent B): from 0 to 10 min, the percentage of B was 5%; from 10 to 47.5, min the percentage of B increased from 5 to 80% B gradient. The flow rate was 1.0 mL/min, and the detection λ was 254 nanometers.

LC-MS analysis was performed on an Agilent (Cernusco sul Naviglio, Milan, Italy) LC-MS ESI-TOF 1260/6230DA instrument operating in negative ionization mode.

The following settings were used: drying gas (nitrogen) 5 L/min, 325 °C; fragmentor voltage 175 V; capillary voltage 3500 V; nebulizer pressure 35 psig. An Agilent Eclipse Plus ODS column (150 × 4.6 mm, 5 µm) (Cernusco sul Naviglio, Milan, Italy) was used. The eluant was the same as described above with a flow rate of 0.4 mL/min.

### 2.2. Preparation of Pomegranate Extracts

Pomegranates were carefully washed with water before use. PPS, obtained after juice preparation by squeezing, were lyophilized and roughly minced by a blender. The resulting pomace (1 g) was extracted with water (10 mL, 100 mg/mL solid-to-liquid ratio) and stirred for 60 min, at RT. The resulting mixture was centrifuged (7000 rpm, 4 °C, 10 min). The supernatant was collected and stored at −25 °C, whereas the solid residue was lyophilized to evaluate the extracted ponderal yield by difference (43% *w*/*w* yield). The PPS were also extracted with ethanol or ethanol/water (6:4 *v*/*v*) under the same conditions described above (36 and 52% *w*/*w* yields, respectively). To complete removal of ethanol before evaluation of the antioxidant and antimicrobial activity, all three extracts were taken to dryness. Then, based on the extraction yields, they were adjusted to the same concentration by adding water.

### 2.3. Preparation of PPS Extract–WPI Samples (POM@WPI)

WPI (500 mg) was added to water (5 mL) and the resulting suspension was brought to pH 12 by dropwise addition of 150 μL of 6 M NaOH and stirred for 30 min at room temperature. Then, the pH was adjusted to 7 adding 200 μL of 6 M HCl followed by the addition of 0.031 mL (corresponding to 0.025 mg of extracts) of the PPS solution. Control sample (sole WPI) was prepared without the PPS extract, and for comparison, 0.031 mL of water, instead of the extract, was added.

### 2.4. DPPH Assay

All the prepared extracts were added (final dose 0.010–0.062 mg/mL) to a 200 μM DPPH ethanolic solution and stirred at room temperature. After 10 min, the absorbance of the solution at 515 nm was measured. The assay was performed also on WPI (final dose 0.2–0.7 mg/mL) and POM@WPI (final dose 0.28–1.4 mg/mL). The results were expressed as EC_50_ values. Experiments were run in triplicate [30,31].

### 2.5. FRAP Assay

The FRAP solution was prepared mixing FeCl_3_ (1.7 mM) and TPTZ (0.83 mM) in 0.3 M acetate buffer (pH 3.6) in a 1:1:10 *v*/*v* ratio. All the extracts were added (final dose 0.0027–0.0125 mg/mL) to a FRAP solution and stirred for 10 min. After this time, the absorbance of the solution at 593 nm was measured.

The same assay was carried out on WPI (final dose 1.5–5 mg/mL) and POM@WPI (final dose 0.07–0.28 mg/mL). Trolox was used as a reference antioxidant. The results were expressed as mg of Trolox/mg of sample. Experiments were run in triplicate [32].

### 2.6. TPC Assay

A solution of Folin–Ciocalteu reagent, 75 g/L Na_2_CO_3_, and water in a 1:3:14 *v*/*v*/*v* ratio was prepared. To the resulting mixture, all the extracts were added at a final dose of 0.0025–0.1 mg/mL and stirred for 30 min at 40 °C. After this time, the absorbance at 765 nm was measured. Gallic acid was used as a reference compound. The results were expressed as mg of gallic acid/mg of sample. Experiments were run in triplicate [33].

### 2.7. Antimicrobial Activity of PPS Extracts

*Pseudomonas* spp. and yeasts were used as target food spoilage to test in vitro the antimicrobial activity of the water/water–ethanol/ethanol PPS extracts. Specifically, the *Pseudomonas* spp., *P. putida*, and *P. fluorescens* were isolated from spoiled mozzarella, and the yeasts were isolated from red pomace, all stored at –20 °C. The two *Pseudomonas* spp. strains were obtained by exponential growth in culture broth, consisting of 5 g/L tryptone, 1 g/L glucose, and 2.5 g/L yeast extract (PC broth, Oxoid), incubated at 25 °C for 24 h. The two strains were prepared mixing 1% of each culture. Both microbial and fungal species were used at concentration of 10^3^ CFU/mL, after diluting the growing cultures with a sterile saline solution (9 g/L NaCl). To test the effectiveness, 5% *w*/*v* of each extract was placed in 20 tubes containing 10 g of PC broth (10 falcons for *Pseudomonas* spp. and 10 for yeasts). All the tubes were incubated at 25 °C for 72 h. At 0, 4, 24, 48, and 72 h of incubation time, an aliquot (1 mL) was taken from each tube for microbiological analyses. The proliferation of microbial and fungal cells was monitored by plate counting. After appropriate dilutions with 0.9% NaCl, the samples were inoculated into Pseudomonas Agar Base (PAB) plates, with a selective supplement of cetrimide fucidin cephaloridine (CFC) for *Pseudomonas* spp. and into Sabouraud Dextrose Agar (SAB) with chloramphenicol to evaluate yeast growth; for both groups, the plates were incubated at 25 °C for 48 h. The pH measurement was performed in duplicate, on two different samples by using a pH meter (Crison, Barcelona, Spain), after proper calibration.

### 2.8. Antimicrobial Activity of Hydroalcoholic PPS Extract

Based on results from in vitro test, the sole hydroalcoholic extract was considered for the subsequent investigation. Different extract concentrations from 1 to 5% *w*/*v* were tested against the *A. acidoterrestris* (DSM 3922, Brunswick, Germany), as typical juice spoilage bacterium. Malt Extract Broth (MEB, Oxoid, Milan, Italy) at 44 °C for 48 h was used to revitalize the microorganism. To test the extract efficacy against *A. acidoterrestris*, 10 mL of MEB was inoculated and put in contact with the extract at 1%, 1.5%, 2%, 2.5%, and 5% *w*/*v*. No extract was added to the control sample. The incubation temperature was 44 °C. Microbiological analyses were performed twice using two different samples. Malt Extract Agar (MEA, Oxoid, Milan, Italy), incubated at 44 °C for 48 h, was adopted.

The antimicrobial efficacy of the hydroalcoholic PPS extract was also tested in apple juice previously inoculated with *A. acidoterrestris* (10^3^ CFU/mL). Three PPS extract concentrations were tested, that is, 1%, 1.5%, and 2% *w*/*v*. Tubes containing the sole apple juice (without any extract and without any inoculation) and tubes with juice inoculated with *A. acidoterrestris* were also prepared and used as the controls. All the tubes were stored at 37 °C for two weeks. During storage, 1 mL aliquot was taken for the analysis. MEA at 44 °C for 48 h was used as selective medium.

### 2.9. Sensory Evaluation

All the apple juice samples were submitted to sensory evaluation. The panel was composed of seven trained panelists, researchers of the Food Department of Foggia University (ages between 28 and 48 years). Although experienced in tasting food products, the panelists were aligned in the sensory attributes and in the scale, in two sessions (1 session/day; 2 h/session). The quality parameters of each sample were color, odor, and overall quality. The scale had 7 points with 1 = lowest score, 7 = highest score, and 4 = threshold for acceptability [34]. To calculate the sensory shelf life of the samples, a mathematical approach was adopted, as reported in another study also dealing with shelf life of apple juice [35]. By fitting the sensory data with a re-parameterized Gompertz equation, the day on which juice remained acceptable was properly calculated.

### 2.10. Acid Degradation of Hydroalcoholic PPS Extract

A total of 5 mL of 4 M HCl was added to a Pyrex tube containing the lyophilized hydroalcoholic PPS extract (50 mg). The mixture was vigorously mixed for 1 min and incubated in an oven at 90 °C [36]. After 24 h, the mixture was cooled at room temperature and the pH of the mixture was adjusted to 2.5 by the addition of 6 M NaOH. The precipitate was collected by centrifugation (7000 rpm, 4 °C, 10 min), whereas the supernatant was recovered and taken to a volume of 10 mL by addition of water. After filtration through a 0.45 µm PVDF filter, the supernatant was analyzed by HPLC, as well as the solid residue after dissolving it in a 10 mL of DMSO/methanol 1:1 *v*/*v*.

### 2.11. Apple Smoothie Browning Inhibition Assay

Red Delicious apples were washed with water and peeled. A total of 5 g of apple cuts was finely blended with a mixer after the addition of 0.1% *w*/*v* PPS extract solution. The resulting mixture was transferred to a watch glass. Blank smoothie samples were prepared in the absence of PPS extract. Color changes were analyzed with a colorimeter [37,38]. Three different experiments were performed, and for each experiment, three different measurements were taken.

The browning index (BI) was calculated as follows:

(1) $$Browning\;index\;\left(BI\right)=\frac{100\left(x-0.31\right)}{0.17}$$

(2) $$x=\frac{\alpha^{*}+1.75L^{*}}{5.645L^{*}+\alpha^{*}-3.012{\;b}^{*}}$$

All values were corrected by subtracting the BI values of the smoothies at time zero.

### 2.12. Apple Cuts’ Browning Inhibition Assay

Nine fresh Red Delicious apples were washed with water, and peeled apples cuts (ca. 500 mg each) were prepared. Subsequently, these pieces were dipped into a WPI or POM@WPI solution prepared as described before (see Section 2.3) and dried at room temperature for 24 h. After carefully removing the coating formed on the apple cuts, changes in color were periodically analyzed with the colorimeter. The browning index (BI) was calculated according to Equations (1) and (2). Three different experiments were performed, and for each experiment, three different measurements were taken. The percentage of inhibition was calculated with respect to the uncoated fruit.

### 2.13. Mass Loss Determination of Apple Cuts

Six freshly peeled Red Delicious apples cuts (ca. 500 mg each) were weighed and then dipped into a WPI or POM@WPI solution prepared as described before (see Section 2.3). After drying in air at room temperature for 24 h, the coating covering the cuts was carefully removed and their weights measured again. As a control, the same experiment was performed on uncoated apple pieces.

### 2.14. Lipid Peroxidation Inhibition Assay in Gouda Cheese (TBARS)

An experimental procedure reported in the literature was followed [39,40,41]. Gouda cheese samples were cut into pieces, with an area of around 4 cm^2^, and heated in an oven at 50 °C after dipping into WPI or POM@WPI solution, together with an untreated control sample. After 12 days, the samples were recovered and minced in a chloroform/methanol mixture 2:1 (60 mL) using a blender. The mixture was then filtered under vacuum using Whatman No. 1 filter paper, and the filtrate was dried using a rotary evaporator. The residue (0.2 g) was homogenized in trichloroacetic acid containing 7.5% *w*/*w* butanol (25 mL). To 5 mL of the resulting mixture, 5 mL of a 0.02 M thiobarbituric acid solution in butanol was added and heated at 95 °C for 2 h. After cooling at room temperature, the absorbance at 539 nm was measured. Experiments were run in triplicate.

### 2.15. Statistical Analysis

To statistically compare experimental data and fitting parameters of antimicrobial assays, the one-way ANOVA analysis was used. A Duncan’s multiple range test, with the option of homogeneous groups (*p* < 0.05), was carried out to determine significant differences among samples. To this aim, STATISTICA 7.1 for Windows (StatSoft, Inc., Tulsa, OK, USA) was adopted.

## 3. Results and Discussion

### 3.1. Preparation of Pomegranate Waste Extracts

In the initial experiments, three different food-grade solvents, that is, water, ethanol, and ethanol/water (6:4 *v*/*v*), were evaluated for the recovery of phenolic compounds from pomegranate wastes. The extraction yields for each solvent were evaluated based on the quantitation of the solid residues after lyophilization as the presence of sugar components hindered the complete drying of the extracts. As shown in Table 1, the highest extraction yields were observed for the hydroalcoholic extract, followed by water, though the difference was not statistically significant, and ethanol, in that order.

DPPH and FRAP assays indicated comparable antioxidant properties for the hydroalcoholic and water extracts, although the first one exhibited a higher TPC value (Table 2). This could be ascribed to the presence of phenol compounds with a poor reducing ability, likely high-molecular-weight compounds that could hardly interact with the DPPH and FRAP reagents under the assay conditions. The results of the DPPH assay in particular were consistent with EC_50_ data reported in the literature for various pomegranate peel extracts ranging from 10 to 50 μg/mL, depending on the extraction conditions and the pomegranate varieties [42].

**Figure 1:**
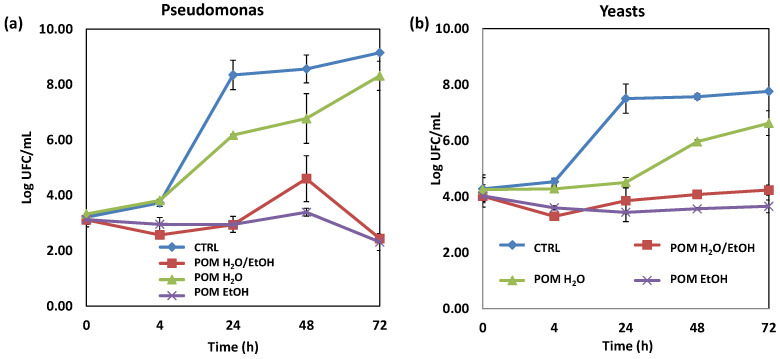
Evolution of *Pseudomonas* spp. (**a**) and yeasts (**b**) with and without the water/water–ethanol/ethanol PPS extracts. Data are presented as means ± SD. CTRL = inoculated sample without extract; POM H_2_O/EtOH = inoculated broth containing water–ethanol PPS extract; POM H_2_O = inoculated broth containing water PPS extract; POM EtOH = inoculated broth containing ethanol PPS extract.

The three PPS extracts were preliminary tested at 5% *w*/*v* against *Pseudomonas* spp. and yeasts to verify their antimicrobial potential on typical food spoilage. Figure 1 shows significant differences between the control sample and the active broths. *Pseudomonas* spp. (Figure 1a) and yeasts (Figure 1b) proliferated very rapidly in the control. Interestingly, differences among the active samples were also observed. When water was used as the extraction solvent, the efficacy was less marked against both *Pseudomonas* spp. and yeasts. In the case of the other two extracts tested, the microbial and fungal cells were significantly delayed and very low cell concentrations were found. The pH remained around 6.5 in the control samples of both *Pseudomonas* spp. and yeasts and around 4.3 in the active samples, due to the presence of pomegranate extract. Yet, such differences in pH values are not per se responsible for the inhibition activity as shown by the control experiments and confirmed by the differences in the antimicrobial power of the extracts of PPS all determining the same pH conditions in the incubation media.

**Figure 2:**
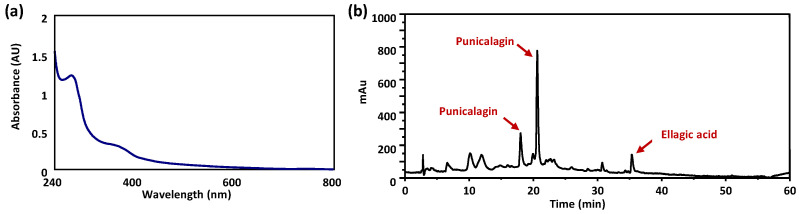
(**a**) UV-Vis spectrum (0.085 mg/mL in water) and (**b**) HPLC profile (10 mg/mL) of PPS hydroalcoholic extract.

This experimental evidence, combined with the extraction yield and the results of the antioxidant assays, allowed selecting the extract obtained with the water/ethanol mixture as the most interesting. Therefore, the hydroalcoholic PPS extract was selected for further investigation. The extract was analyzed by UV-Vis spectroscopy and HPLC. The UV-Vis spectrum featured two absorption maxima at 260 and 375 nm (Figure 2a), indicative of the presence of ellagic acid and/or ellagitannins.

HPLC analysis (Figure 2b) showed, among other things, the presence of three main peaks eluted at ca. 19, 21, and 36 min; they were identified by LC-MS analysis as punicalagin isomers and ellagic acid, in that order.

The content of ellagic acid and punicalagin isomers was estimated at 5% *w*/*w* by HPLC analysis, taking ellagic acid as reference; this value that was also confirmed by UV-Vis analysis by the use of an ellagic acid calibration curve. Ellagitannins are not amenable to be analyzed by conventional chromatographic methodologies like LC-MS or LC-UV due to their polymeric nature. Therefore, alternative methods are usually adopted involving the chemical degradation and analysis of the resulting products. In this case, acid degradation of the lyophilized hydroalcoholic extract allowed us to confirm the presence of hydrolysable ellagitannins and to estimate a 16% *w*/*w* content as ellagic acid equivalents.

**Figure 3:**
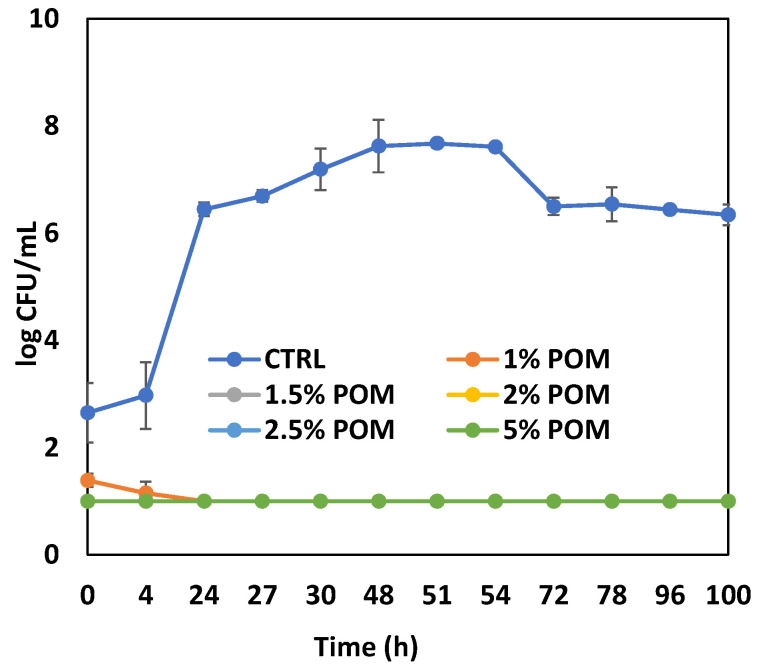
Evolution of *Alicyclobacillus acidoterrestris* in the absence or in the presence of different amounts (1, 1.5, 2, 2.5, and 5% *w*/*v*) of the water/ethanol PPS extract. Data are presented as means ± standard deviations. CTRL = inoculated broth without extract; 1, 1.5, 2, 2.5, and 5% POM = inoculated broths containing 1, 1.5, 2, 2.5, and 5% PPS extract.

The water/ethanol extract was then tested in vitro against *A. acidoterrestris* as a typical juice spoilage bacterium that is able to survive at acidic pHs at values as low as pH 3.7 [43]. To this aim, different concentrations of extract, from 1% to 5% *w*/*v*, were tested. Figure 3 shows the results for the control and samples of PPS hydroalcoholic extract at all concentrations. As can be seen, in the control, the *A. acidoterrestris* reached a concentration of 10^8^ CFU/mL within about 2 days, whereas in the sample with the extract, the microorganism growth was completely inhibited. It appears that a 2% concentration of the extract was enough to produce a complete inhibition. The pH remained around 5.5 in the control samples and around 4.2 in the samples with the extract. This result is of particular interest as the PPS extract had never been tested on this type of bacterium and, in this respect, integrates the scientific literature on the antimicrobial activity of these extracts. For example, it was demonstrated that the extract obtained from pomegranate peel, in addition to an excellent antioxidant activity, showed a strong antimicrobial action with a minimum inhibitory concentration of 0.01% against *Staphylococcus aureus* and *Bacillus cereus* [18]; in fact, its addition to chicken meat-based products was effective in controlling oxidative rancidity, as well as prolonging the shelf life. The improvement of the quality and shelf life of other kinds of food was also recently reported [44]. Similarly to our study, pomegranate peels were preliminarily subjected to extraction using different solvents (water, ethanol, and methanol alone or in combination with water) and subsequently evaluated for the antibacterial activity of the extract against different pathogens, such as *Staphylococcus aureus*, *Enterobacter aerogenes*, *Salmonella typhi*, and *Klebsiella pneumoniae* [45]. The results demonstrated that the extracts possessed notable antibacterial activity against all the tested pathogens. Several studies showed a broad-spectrum antimicrobial activity of pomegranate extracts against both *Gram*+ and *Gram*− bacteria and demonstrated that the antimicrobial activity of the extracts obtained from pomegranate peel was stronger than that of extracts obtained from other parts of the fruit (seeds, pulp) [17]. This type of extract was shown to be a rich source of antibacterial constituents that can reduce the emergence of bacterial microbes generally involved in food spoilage and foodborne diseases [46].

### 3.2. Evaluation of the Antimicrobial Activity of PPS Hydroalcoholic Extract in Apple Juice

**Figure 4:**
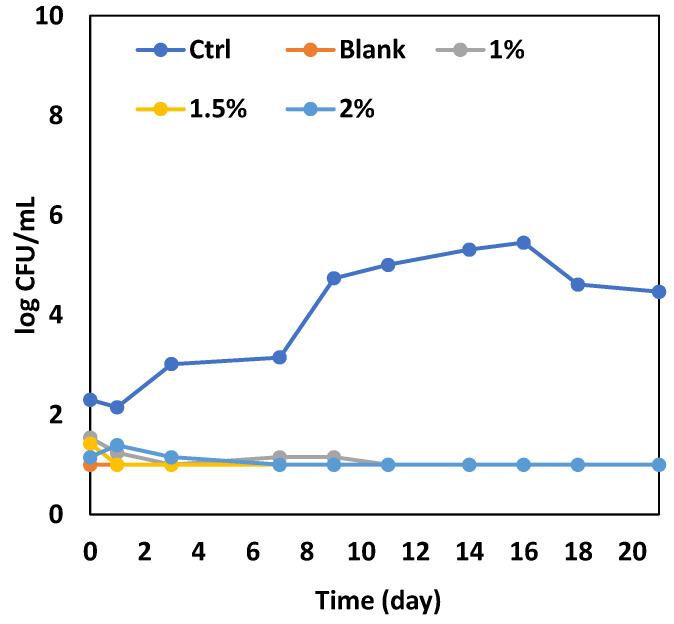
Evolution of *A. acidoterrestris* inoculated in apple juice with and without the PPS hydroalcoholic extract at different amounts (1, 1.5, and 2% *w*/*w*). Data are reported as means ± standard deviations. Ctrl = inoculated juice without extract; Blank = juice without inoculation and extract; 1, 1.50, and 2% = inoculated juices containing 1, 1.50, and 2% *w*/*w* PPS extract.

Based on the results obtained from the in vitro test, the three lowest concentrations of the hydroalcoholic extract (1%, 1.5% and 2% *w*/*v*) were used in apple juice previously inoculated by *A. acidoterrestris*. Apple juice was selected as a specific food matrix for *A. acidoterrestris* proliferation since this bacterium requires a carbohydrate-rich medium and apple juice mainly contains sugars and carbohydrate fibers. In this test, both microbiological and sensory quality were monitored during a storage period of approximately 3 weeks, to verify the extract effectiveness under real working conditions. The storage period of apple juice depends on the conditions, such as temperature and package opening. In fact, packaged apple juice under refrigerated storage conditions could be stored for several days, whereas, if unpackaged, it lasts a few days. In our case, the product was inoculated and stored under thermal abuse to favor microbial growth and, therefore, 3 weeks was a proper storage time for observation. As can be seen from Figure 4, all the extract concentrations were effective and promoted a total microbial inhibition. The two control samples were in line with what one would expect. Indeed, in the blank control juice, the microorganism never grew, whereas in the inoculated control sample, a microbial proliferation rapidly occurred. Similar experimental evidence was also recorded with a sunflower meal ethanol solute when tested against *A. acidoterrestris* [35]. The pH was around 3.5 in the control juice and around 3.3 in the fortified samples. Regarding the sensory quality of juice during storage, the blank sample never reached any sensory threshold because it remained acceptable in terms of both odor and color for the entire observation period and, therefore, it demonstrated an overall quality for more than 3 weeks. The other inoculated samples lasted a different number of days, depending on the addition of the extract (Table 3). The inoculated control juice lasted less than 12 days, mainly due to unacceptable odor changes [43], whereas the three samples with the PPS extract remained acceptable for 17–20 days, thus demonstrating that bacterial inhibition also allowed maintaining a better sensory overall quality [35].

### 3.3. Evaluation of the Enzymatic Browning Inhibition by PPS Hydroalcoholic Extract in Apple Smoothies

**Figure 5:**
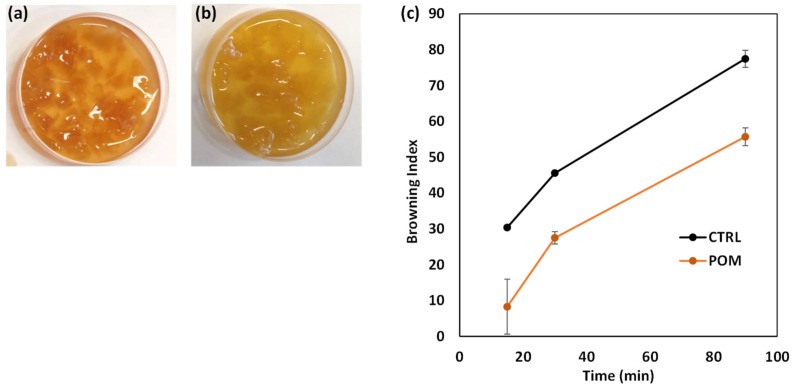
Apple smoothies (**a**) without and (**b**) with 0.1% *w*/*v* PPS hydroalcoholic extract at 30 min. (**c**) Browning index for apple smoothies with or without the extract with respect to the initial value. Mean ± SD values of three experiments are reported (three different measurements were taken during each experiment).

Browning of fresh-cut fruits represents an issue of concern because of the loss of the commercial value of the products. Control of this process is generally implemented by use of antioxidant compounds preferably of a natural origin [47]. On this basis, experiments were directed at evaluating the ability of the PPS hydroalcoholic extract to inhibit enzymatic browning in apple smoothies. The aim of this experiment was to assess how effectively the extract could prevent oxidation, thus preserving the quality and freshness of the smoothies. Apples were finely grinded with a blender in the presence or absence of 0.1% *w*/*v* PPS extract, and the changes in color were periodically analyzed with a colorimeter. The effect of the presence of the extract was well apparent after 30 min (Figure 5a,b), at which time the browning index for the smoothies containing the extract was around 27.5 ± 1.5, whereas for the untreated, it was around 45.5 ± 0.3 (Figure 5c). The effect was observed also in the first stages of the process when the rate of the browning development was very fast.

### 3.4. Combination of PPS Hydroalcoholic Extract with WPI

Given the promising results in the model food systems described above, the possible use of a PPS extract in food packaging applications was evaluated. In particular, the possible combination of the extract with WPI was considered, given the excellent ability of this proteinaceous matrix to provide edible coatings [48]. Initially, the optimization of the conditions for WPI denaturation, a parameter that critically affects the film and coating-forming ability of these proteins, was run. The effects of pH, temperature, and concentration were systematically explored. The best conditions were the stirring of a 10% *w*/*w* suspension of WPI in water taken to pH 12 over 30 min. For the incorporation of a PPS hydroalcoholic extract, in a first series of experiments, the remaining aqueous solution after the removal of ethanol with the aid of a rotatory evaporator was added to a denatured WPI suspension at a 5% *w*/*w* final concentration with respect to WPI to give POM@WPI.

The addition of this extract containing ellagic acid and related polyphenols, as described under Section 3.1, was expected to confer WPI antioxidant properties. To assess this, DPPH and FRAP assays were run on the POM@WPI mixture. Yet, no appreciable activity was observed in either assay. This can be likely ascribed to a significant oxidation of the polyphenol components of the extract when exposed to air at the alkaline pH conditions chosen for the WPI denaturation. Based on these considerations, in further experiments the pH of the WPI denaturation solution was adjusted to 7 before the addition of the PPS extract and the antioxidant properties of the POM@WPI were evaluated again against those of the WPI solution without the addition of the extract. In the DPPH assay, the POM@WPI exhibited EC_50_ values about 5-fold lower than the WPI alone (Table 4). An increase in the reducing properties due to the presence of the extract was observed also in the case of the FRAP assay. Based on the amount of extract present in the final material that was 5% *w*/*w* and on the values reported in Table 2, one should obtain an EC_50_ value and mg Trolox/mg of a sample equal to 0.0400 and 0.165, respectively, for POM@WPI. Notably, these values were of the same order of magnitude as those that were experimentally determined, thus providing evidence that the polyphenolic components of the extract maintain most of their antioxidant potential in the final material.

### 3.5. Food Applications of POM@WPI

For a preliminary evaluation of POM@WPI in a food-coating application, different food matrices, specifically cheese and apples, that are particularly prone to deterioration were selected. The experiments were aimed at providing a preliminary evaluation of the effectiveness of this coating in food preservation.

**Figure 6:**
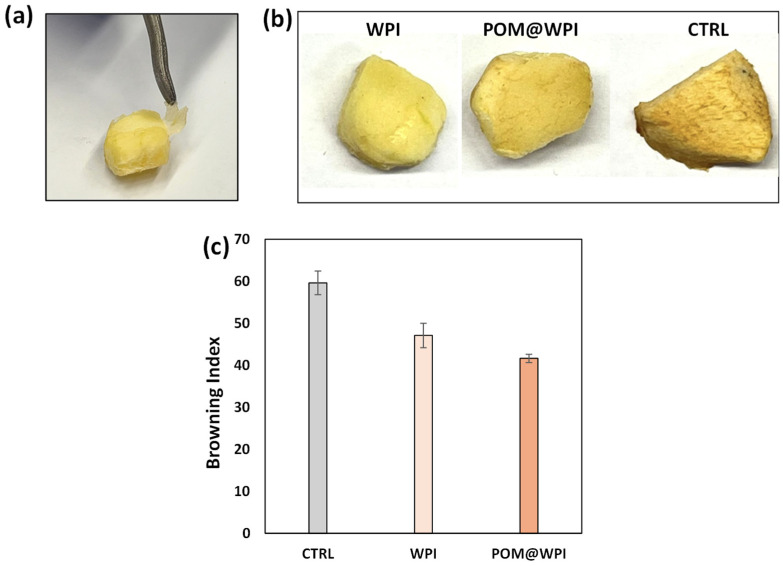
(**a**) Picture of WPI film removal from the apple cuts; (**b**) pictures of coated apple cuts; (**c**) browning index of apple cuts coated with WPI and POM@WPI after 24 h. Mean ± SD values of three experiments are reported (three different measurements were taken during each experiment).

Apple cuts were dipped into the POM@WPI or WPI solutions and then air-dried for 24 h. An uncoated sample was also evaluated as a control. Notably, the removal of the WPI film from the apple cuts proved very easy (Figure 6a). The mass loss of the POM@WPI- and WPI-coated apple cuts was determined after the removal of the coating in comparison with the untreated sample. The POM@WPI- and WPI-coated samples gave values of 64 ± 6% and 58 ± 3%, respectively, vs. values of 75 ± 4% for the control sample (Figure 6b). No significant differences were observed between the WPI- and POM@WPI-covered samples, indicating that the decreased weight loss was due to the barrier action of the WPI and that the extract did not affect such property. On the other hand, the browning process was delayed as determined by the browning index of 41 ± 2 for the POM@WPI and 48 ± 1 for WPI. This result confirms the enzymatic browning inhibition ability of the PPS extract, which significantly contributed to the oxygen barrier properties of the WPI coating (Figure 6c).

**Figure 7:**
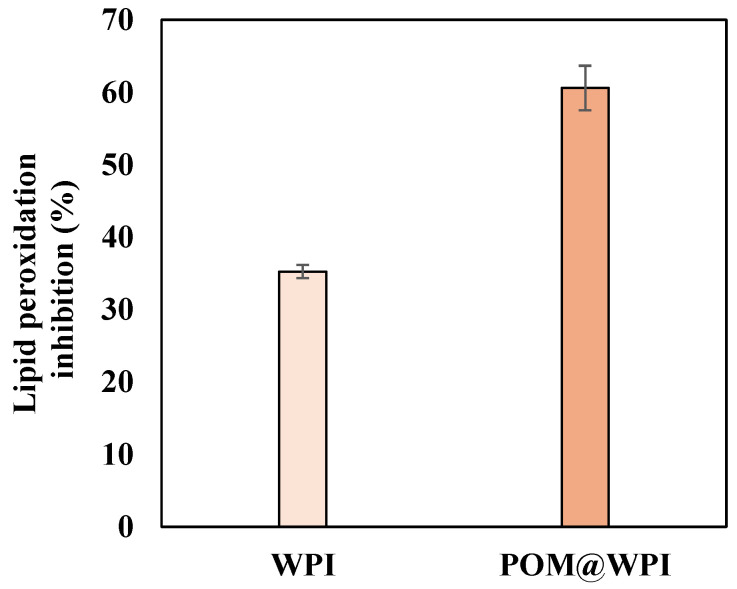
Lipid peroxidation inhibition by WPI and POM@WPI coatings on Gouda cheese samples after exposure to accelerated aging over 12 days. Data reported are the mean ± SD values from at least three experiments.

To test the potential of POM@WPI coating to prevent the lipid peroxidation process that is responsible for food degradation, cheese was chosen as a suitable food matrix. Pieces of Gouda cheese were coated with POM@WPI or with WPI and subjected to accelerated aging, following a procedure previously developed [40,41] involving storage in an oven at 50 °C. Monitoring of the peroxidation of coated Gouda cheese against uncoated pieces by a thiobarbituric acid reactive substances (TBARS) assay allowed us to select a period of 12 days as optimal for evaluating the process. A decrease in the extent of lipid peroxidation was observed with both the POM@WPI- and the WPI-treated samples compared to the control cheese. Notably, the POM@WPI proved about 2-fold more efficient than WPI in preserving the cheese, with a 60 ± 3% inhibition vs. a 35.2 ± 0.9 % inhibition of WPI (Figure 7). This result confirmed that the PPS extract was able to impart its antioxidant properties to the WPI coating. It can be concluded that WPI is able to retard lipid peroxidation but the presence of the extract in the POM@WPI remarkably enhances the inhibitory effect.

## 4. Conclusions

Though the antioxidant and antimicrobial activity of pomegranate, as well as of PPS extracts, was already described in the literature, the present study provides a valuable contribution for what concerns antimicrobial activity against typical food spoilage bacteria and yeasts. Indeed, the effects on the acidophilic *A. acidoterrestris*, whose contamination of apple juices represents a considerable threat to the juice industry, were tested on a real food sample with very encouraging results.

In addition, the possibility to exploit the PPS extract for implementation of an active food coating using whey proteins, the most abundant low-cost by-product of dairy industries, represents an interesting outcome of this study. The film-forming properties of WPI allowed us to obtain the deposition of a coating on the food to be preserved by simple dipping. On the other hand, the removal of the coating from the food proved to be extremely simple. The results of the experiments run on the selected food models confirmed that the addition of the PPS extract provided additional functional properties to the WPI coatings, already endowed with preserving activity mainly due to their gas barrier action. Indeed, the POM@WPI coatings exerted efficient antioxidant protection and enzymatic browning inhibition activity on matrices such as cheese and cut fruit, respectively. Considering that WPI films and coatings were evaluated for edible packaging due to the high biocompatibility [48], the results of the present study open new perspectives on food preservation strategies highlighting the potential of PPS extract for the implementation of a sustainable WPI-based functional packaging. Application of the results of this study to food packaging technology would require further investigation to improve the mechanical properties of the coatings and their resistance over the shelf life of the food.

## Figures and Tables

**Table 1:** Extraction yields ± SD of PPS with various solvents. Different letters denote statistically significant differences (*p* < 0.1).

Solvent	Extraction Yield (% *w*/*w*) *
H_2_O	43 ± 5% ^ab^
Ethanol/H_2_O	52 ± 3% ^b^
Ethanol	36 ± 2% ^a^

* Calculated by difference with respect to the solid residue.

**Table 2:** Antioxidant properties and total phenol content of PPS extracts in various solvents.

Solvent	EC_50_ (mg/mL) DPPH	mg of Trolox/ mg of Sample FRAP	mg of Gallic Acid/ mg of Sample TPC
H_2_O	0.022 ± 0.001 ^a^	0.35 ± 0.01 ^a^	0.16 ± 0.01 ^a^
Ethanol/H_2_O	0.020 ± 0.001 ^a^	0.33 ± 0.02 ^a^	0.24 ± 0.04 ^b^
Ethanol	0.049 ± 0.001 ^b^	0.26 ± 0.02 ^b^	0.087 ± 0.02 ^b^

Data are the mean ± SD values from at least three experiments. For DPPH and FRAP assays, different letters denote statistically significant differences (*p* < 0.05); for TPC assay, different letters denote statistically significant differences (*p* < 0.1).

**Table 3:** Values of sensory shelf life of juice calculated by the fitting procedure.

Sample	Overall Quality Fitting Parameter (Day)
Blank juice	>25
Inoculated control juice	11.26 ± 0.93 ^c^
Juice with 1% extract	20.02 ± 1.21 ^a^
Juice with 1.5% extract	17.77 ± 0.92 ^b^
Juice with 2% extract	17.14 ± 0.64 ^b^

Different letters denote statistically significant differences (*p* < 0.05).

**Table 4:** Antioxidant properties of POM@WPI and WPI.

Sample	EC_50_ (mg/mL) DPPH *	mg Trolox/mg of Sample FRAP
POM@WPI	0.71 ± 0.1	0.0185 ± 0.0008
WPI	3.9 ± 0.6	<0.0005

Data are the mean ± SD values from at least three experiments. * Statistically significant differences (*p* < 0.1).

## Data Availability

The original contributions presented in the study are included in the article; further inquiries can be directed to the corresponding author/s.
